# Electric and Photocatalytic Properties of Graphene Oxide Depending on the Degree of Its Reduction

**DOI:** 10.3390/nano10112313

**Published:** 2020-11-22

**Authors:** László Péter Bakos, Lőrinc Sárvári, Krisztina László, János Mizsei, Zoltán Kónya, Gyula Halasi, Klára Hernádi, Anna Szabó, Dániel Berkesi, István Bakos, Imre Miklós Szilágyi

**Affiliations:** 1 Department of Inorganic and Analytical Chemistry, Budapest University of Technology and Economics, Szent Gellért tér 4, H-1111 Budapest, Hungary; laszlobakos@mail.bme.hu (L.P.B.); sarvari.lorinc@gmail.com (L.S.); 2 Department of Physical Chemistry and Materials Science, Budapest University of Technology and Economics, Budafoki út 8. F. I. building, H–1111 Budapest, Hungary; klaszlo@mail.bme.hu; 3 Department of Electron Devices, Budapest University of Technology and Economics, H-1117 Budapest, Hungary; mizsei@eet.bme.hu; 4 Department of Applied and Environmental Chemistry, University of Szeged, Rerrich Béla tér 1, H-6720 Szeged, Hungary; konya@chem.u-szeged.hu (Z.K.); halasigy@chem.u-szeged.hu (G.H.); hernadi@chem.u-szeged.hu (K.H.); szabo.anna@chem.u-szeged.hu (A.S.); daniel.berkesi@chem.u-szeged.hu (D.B.); 5 Institute of Materials and Environmental Chemistry, Research Centre for Natural Sciences, Magyar tudósok körútja 2, H-1117 Budapest, Hungary; bakos.istvan@ttk.mta.hu

**Keywords:** graphene oxide, reduced graphene oxide, photocatalysis, electrical resistance, cyclic voltammetry

## Abstract

When graphene oxide is reduced, the functional groups are released and the structure becomes more ordered. The degree of reduction might be tunable with the process parameters. In our work, graphene oxide is prepared and the effect of thermal and chemical reduction is investigated. The samples are characterized with TG/DTA-MS, SEM-EDX, TEM, XPS, ATR-FTIR, Raman spectroscopy and XRD. Their electrical resistance, cyclic voltammetry and photocatalytic activity data are investigated. The conductivity can be varied by several orders of magnitude, offering a tool to match its electrical properties to certain applications. Low temperature reduction in air offers a material with the highest capacitance, which might be used in supercapacitors. The bare graphene oxide has considerably larger photocatalytic activity than P25 TiO_2_. Reduction decreases the activity, meaning that reduced graphene oxide can be used as an electron sink in composite photocatalysts, but does not contribute to the photocatalytic activity by itself.

## 1. Introduction

The graphene oxide (GO) is a widely studied nanostructured material, being the semiconducting derivative of the graphene. There are many methods to produce graphene oxide, and one of the most frequently used is the improved Hummers method, during which graphite is oxidized and exfoliated [1,2,3]. To partially restore the graphene structure, reduction can be utilized, chemically (usually with ascorbic acid, hydrazine or NaBH_4_) or thermally, and the resulting product is the reduced graphene oxide (RGO) [4,5,6,7,8,9,10]. By modifying the parameters (e.g., time, temperature, reagents) of these processes, the degree of reduction can be varied, which significantly influences the optical and electronic properties, because they mainly depend on the type and amount of the functional groups and heteroatoms present on the graphene, and the saturation of bonds. Regarding the n/p-type semiconductor characteristics of the RGO, if mostly electron-withdrawing (e.g., carboxyl, carbonyl) groups are present, the RGO will be p-type, and if electron-donating groups dominate (e.g., ether, epoxide), the result will be n-type RGO [11,12,13,14].

The conductivity of GO can be varied from the semiconductor GO to the highly conductive reduced GO. This makes them promising candidate materials for electrodes and for other applications, where it is important that their resistance values can be fitted properly. They can also be used in electrochemical energy storage as supercapacitors, i.e., the theoretical capacitance of monolayer graphene can be as high as 550 F g^−1^ [15,16,17,18].

GO and RGO, like many other carbon nanostructures, are widely studied in photocatalysis as co-catalysts of semiconductors (e.g., TiO_2_, ZnO and Cu_2_O). Because of their beneficial properties, they improve the charge separation of the photogenerated electron–hole pairs, and sensitize the metal oxide to visible light through the metal–oxygen–carbon bond [19,20,21,22,23,24,25]. Although hundreds of articles and many reviews investigated the photocatalytic GO-based nanocomposites, only a few papers studied the photocatalytic activity of the bare GO and RGO. Graphene oxide has innate photocatalytic activity, similarly to many semiconducting carbon materials, although it has been studied in only a few reports in its bare form [26,27,28,29]. By utilizing reduction to the GO to varying degrees, the functional groups and heteroatoms can be partially removed, influencing the semiconductor properties, on which the photocatalytic activity heavily depends. RGO is also widely used in composite photocatalysts; however, there is little knowledge of its inherent photocatalytic performance.

In our study, the effect of thermal reduction on the graphene oxide was investigated. First, graphene oxide was prepared by the improved Hummers’ method, and different heat treatments (200, 300, and 900 °C) and atmospheres (air, nitrogen and argon/hydrogen) were used to reduce it. The reduction process was followed by thermogravimetry/differential thermal analysis coupled with mass spectrometry (TG/DTA-MS). As well as this, one specimen was made by the chemical reduction of GO with ascorbic acid for reference. The samples were characterized with transmission and scanning electron microscopy (TEM and SEM) and energy dispersive X-ray spectroscopy (EDX), X-ray photoelectron spectroscopy (XPS), attenuated total reflection Fourier-Transform infrared (ATR-FTIR) and Raman spectroscopy and X-ray diffraction (XRD). To test for applications, their electrical resistances, cyclic voltammetric curves and photocatalytic activities were measured.

## 2. Materials and Methods

### 2.1. Preparation of the Graphene Oxide

Graphene oxide (GO) was prepared according to the modified Hummers’ method [30]. For 5 g natural graphite powder (Graphite Tyn, Taiyuan, China), 200 cm^3^ cc. H_2_SO_4_, 25 cm^3^ cc. H_3_PO_4_, and 25 g solid KMnO_4_ were added (all reagents except the graphite were bought from Sigma-Aldrich, Darmstadt, Germany). The mixture was stirred for 15 min, while the temperature was held below 40 °C using water cooling. Afterwards, it was diluted with 500 cm^3^ distilled water and cooled with ice, and the unreacted permanganate was neutralized with hydrogen peroxide (35%). After 12 h of sedimentation, the mixture was centrifuged at 6500 rpm for 5 min (Jouan BR4i Multifunction Centrifuge, Thermo Scientific, Waltham, MA, USA), then the upper part of the suspension was decanted, 1 M HCl was poured in, and this process was repeated five times. In the next step, the HCl was changed to distilled water with the same method, but, by utilizing 30, 60 and 120 min centrifuge times and at 9500 rpm, this time the decanted brown liquid was collected and reused after each step. The remaining black, unreacted graphite was removed. In the end, the viscous black graphene oxide suspension remained. For the further experiments, GO suspension was dried in an oven at 120 °C overnight, resulting in dried-out GO flakes.

### 2.2. Reduction of the Graphene Oxide

The graphene oxide was thermally reduced in a TA Instr. SDT 2960 simultaneous TG/DTA device (New Castle, DE, USA), in an open platinum crucible under 130 cm^3^ min^−1^ gas flow. Three different atmospheres were investigated: air (oxidizing), nitrogen (inert), 95%/5% argon–hydrogen mixture (reducing). The annealing conditions were the following:

- 2.5 mg GO, 10 °C min^−1^ to 900 °C, (samples: GO-air 900°C, GO-N2 900°C, GO-H2 900°C);
- 8 mg GO, 10 °C min^−1^ to 200 °C, then 30 min isotherm section, (samples: GO-air 200°C, GO-N2 200°C, GO-H2 200°C);
- 8 mg GO, 10 °C min^−1^ to 200 °C, then 5 °C min^−1^ to 300 °C, (samples: GO-air 300°C, GO-N2 300°C, GO-H2 300°C).

To obtain chemically reduced graphene oxide (GO AA), 10 mg GO and 100 mg L-ascorbic acid were dispersed in 50 cm^3^ distilled water in an ultrasonic bath, then the suspension was stirred for 3 h at room temperature [4]. The product then was centrifuged for 5 min at 6500 RPM several times, and finally dried in a heating cabinet at 120 °C for a night.

### 2.3. Characterization Methods

TG/DTA-MS measurements were conducted concurrently with the thermal reduction on the same device. The evolved gases during the heating were analyzed by using Balzers Instruments Thermostar GSD 200T quadruple mass spectrometer (MS) (Balzers, Liechtenstein) coupled to the TG/DTA instrument. The ion current of the species between 1 and 64 m/z were followed. SEM-EDX data were obtained by a JEOL JSM-5500LV scanning electron microscope (Tokyo, Japan) using 20 kV accelerating voltage in high vacuum mode. The average elemental composition from EDX data was calculated from three different measurements on each sample. TEM images were taken on a FEI Tecnai G2 20-TWIN transmission electron microscope (Hillsboro, OR, USA). The XPS spectra were made using a SPECS instrument equipped with a Phoibos 150 MCD-9 analyzer (Berlin, Germany). The X-ray source 150 W (14 kV) was Al K α radiation at 40 eV pass energy and 0.3 s dwell time. CasaXPS software was used for data evaluation. The samples were pressed onto the surface of indium foil in order to prevent the additional increase in the C1s intensity. The ATR-FTIR were carried out on a Bruker Tensor 37 with a Specac Golden Gate ATR accessory (Billerica, MA, USA). Raman spectra were recorded on Jobin Yvon Labram Raman instrument equipped with an Olympus BX41 microscope using green laser with a wavelength of 532 nm (Edison, NJ, USA). XRD measurements were made on a PANalytical X’Pert Pro MPD X-ray diffractometer (Malvern, UK) with Cu Kα radiation.

Electrical resistance was measured by a Keithley 616 Digital Electrometer (Cleveland, OH, USA) in a custom-made measurement setup. The samples were put in a small screw hole in a polymer block, a screw was used to secure the samples to a copper plate on the other side, and the screw and plate served as electrodes, both of which were sputtered with 50 nm Au/Pd (Polaron Sputter Coater SC7020, Laughton, UK) (measurement setup: Figure S1). For the cyclic voltammetry (CV) tests, a suspension was made from the samples with water–isopropyl–alcohol (4:1) mixture (1 cm^3^ for 1 mg sample), 4 µL Nafion solution (DuPont™ Nafion^®^ PFSA Polymer Dispersions DE 520, Wilmington, DE, USA) was also given to 1 cm^3^ suspension, and it was ultrasonicated for 30 min. A total of 15 µL from this suspension was then dried on a glassy carbon electrode (d = 5 mm). The measurements took place in a three-compartment electrochemical cell, and the reference electrode was hydrogen electrode, immersed in the same solution as the working electrode. The compartment of the working electrode was purged with argon gas (99.9995%) to remove air. The auxiliary electrode was a platinum plate. For the experiments, 0.5 M H_2_SO_4_ solution was used, between 80 and 850 mV potential limits; the sweep rate was 50 mV s^−1^. Photocatalysis was measured by putting 1 mg of the graphene oxide and reduced graphene oxide samples in cuvettes with 3 cm^3^ aqueous solution of methyl orange (c_0_ = 4*10^−5^ M). The cuvettes were sealed, and were kept in the dark for 24 h so the adsorption equilibrium could occur. The next day, the cuvettes were placed between two Osram 18 W blacklight lamps (Munich, Germany) (spectrum in Figure S2); the distance between them was 5 cm. After the irradiation started, the absorbance was measured every half hour for four hours using a Jasco V-550 UV-VIS spectrophotometer (Tokyo, Japan). The decomposition of the dye was followed by its most intensive peak at 464 nm.

## 3. Results and Discussion

### 3.1. Characterization

**Figure 1:**
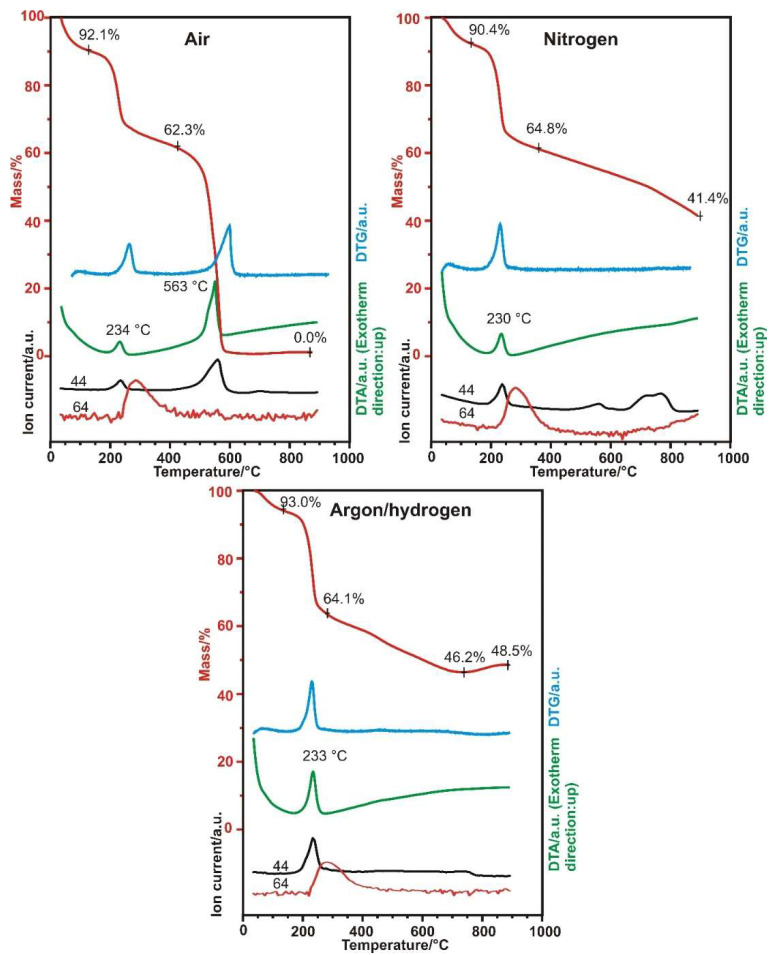
TG/DTA-MS measurements of the GO in different atmospheres to 900 °C (red: TG, blue: DTG, green: DTA, ion currents: black: 44 m/z, red: 64 m/z).

**Figure 2:**
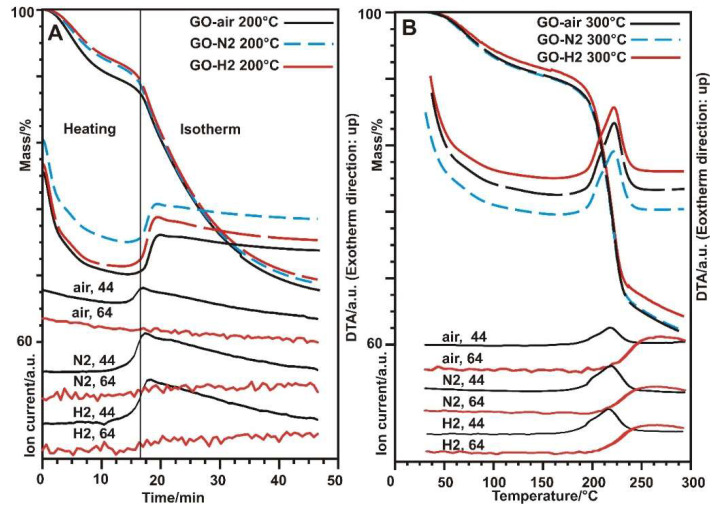
TG/DTA-MS measurements of the GO in different atmospheres to 200 °C (**A**) and 300 °C (**B**) (black: GO-air, blue: GO-N2, red: GO-H2, ion currents: black: 44 m/z, red: 64 m/z).

According to the TG/DTA-MS results in Figure 1 and Figure 2, the different atmospheres have a noticeable effect on the behavior of the GO. In the mass spectra, two ion currents belonging to CO_2_ (at m/z = 44) and SO_2_ (at m/z = 64) are shown. Around 200 °C, an exothermic decomposition step started, with the secession of CO_2_ from the oxygen containing functional groups. No SO_2_ evolution was detected when heated at this temperature and kept isotherm, only when heated at 300 °C. The SO_2_ molecule came from the sulfate ester groups on the surface of the GO, which were created during the synthesis process [31]. In air at 563 °C, the sample started to burn, and fully burnt out at 600 °C. In nitrogen, the sample decomposed steadily, while in hydrogen a slight mass increase (~2%) happened above 700 °C, which is likely to result from the hydrogenation of the aromatic system [32]. For the heat treatments at 200 and 300 °C, an 8 mg sample was used to obtain more product, but the decomposition of the functional groups was very violent, causing the samples to explode out of the crucible, so the heating rate above 200 °C had to be lowered to 5 °C min^−1^ [33].

**Figure 3:**
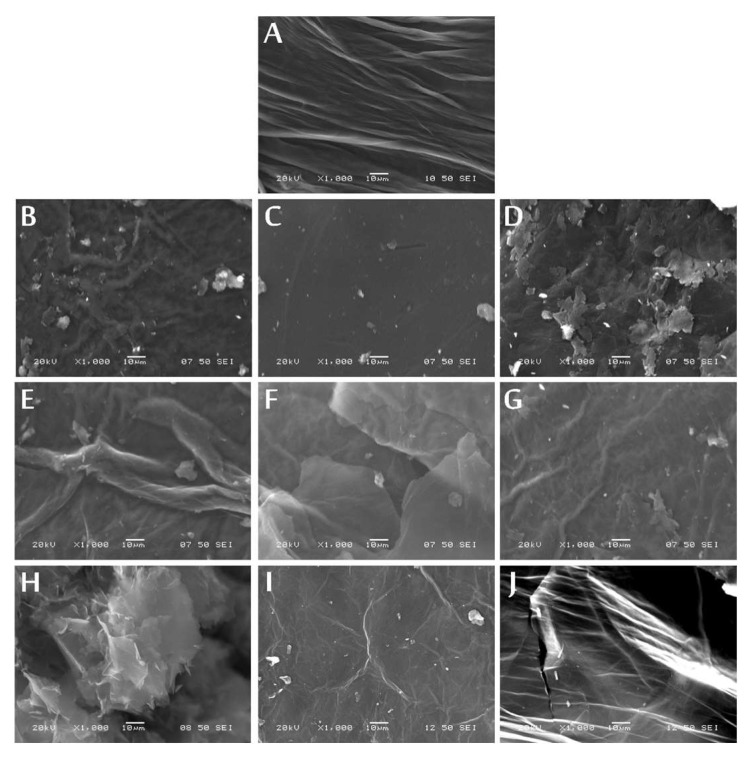
SEM images of the GO (**A**), GO-air 200°C (**B**), GO-N2 200°C (**C**), GO-H2 200°C (**D**), GO-air 300°C (**E**), GO-N2 300°C (**F**), GO-H2 300°C (**G**), GO AA (**H**), GO-N2 900°C (**I**) and GO-H2 900°C (**J**).

**Figure 4:**
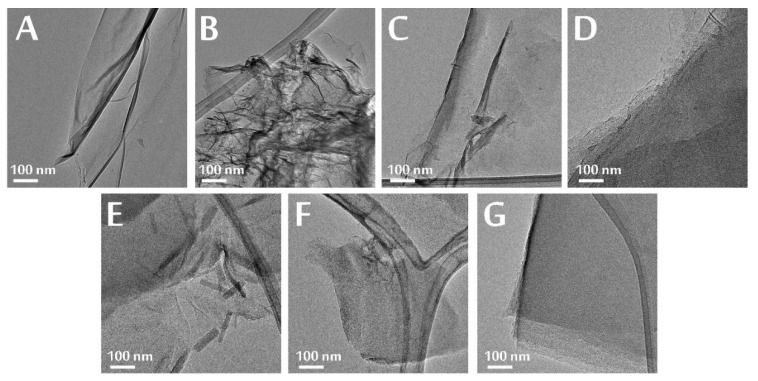
TEM images of the GO (**A**), GO AA (**B**), GO-air 200°C (**C**), GO-air 300°C (**D**), GO-N2 200°C (**E**), GO-N2 300°C (**F**) and GO-N2 900°C (**G**).

In the SEM and TEM images in Figure 3 and Figure 4, the layered structure of the samples can be seen. The heat treatments resulted in concise structures, and in the case of the SEM images, separate sheets were not visible, only agglomerates, which were, however, dismantled by sample preparation for the TEM, revealing the individual plates again. Reduction with ascorbic acid caused fragmentation (Figure 3H and Figure 4B), because of the ultrasonication used to disperse the GO, but the plate-like structure was maintained.

**Figure 5:**
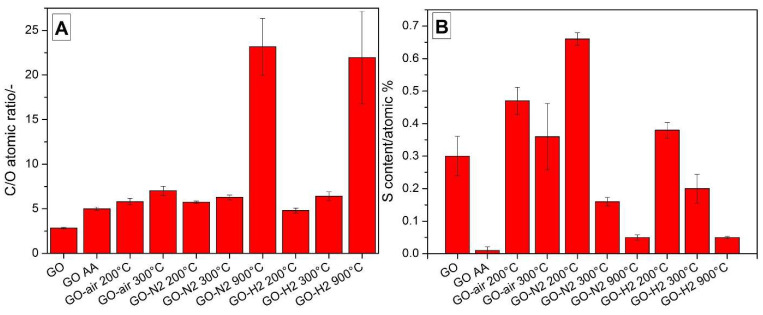
C/O ratio (**A**) and sulfur content (**B**) of the samples from EDX measurements.

From the EDX spectra, the C/O ratio (Figure 5A) for the GO is around 3, meaning that the GO is considerably oxidized. After thermal reduction at 200 °C in all atmospheres, this ratio doubled, and increased further at 300 °C due to further reduction. At 900 °C in nitrogen and argon/hydrogen atmospheres, the remaining oxygen content was very low. In terms of C/O ratio, the sample chemically reduced with ascorbic acid is similar to the 200 °C thermal reduction. The sulfur content (Figure 5B) increased when heated to 200 °C, because only the oxygen-containing functional groups left the samples, as seen in the TG/DTA-MS results, and decreased with further annealing in all atmospheres, since the organosulfates decomposed as well [34]. In case of the GO AA, the sulfur content was negligible after the reduction with ascorbic acid [1].

The XPS results are shown in Table 1, the survey spectra and the deconvoluted C1s and O1s peaks are presented in Figures S3–S5. After heat treatments, more carbon was present as sp2 instead of sp3, and the amount bonded to oxygen as C-O or C=O decreased as well, which indicates the restoration of the graphene structure. GO AA has a larger amount of sp3 carbon, due to the fragmentation of the plates caused by the ultrasonic treatment. The oxygen content decreased significantly with heat treatments; the largest decrease was observed in hydrogen at 300 °C, while it decreased less in air and the least in nitrogen. In the case of the sample heated in nitrogen at 300 °C, nitrogen was incorporated in the structure because its peak appeared in the XPS spectrum (Figure S6). The examined samples had sulfur content similar to the EDX values, and sulfur was not detectable for the GO AA with XPS as well.

**Figure 6:**
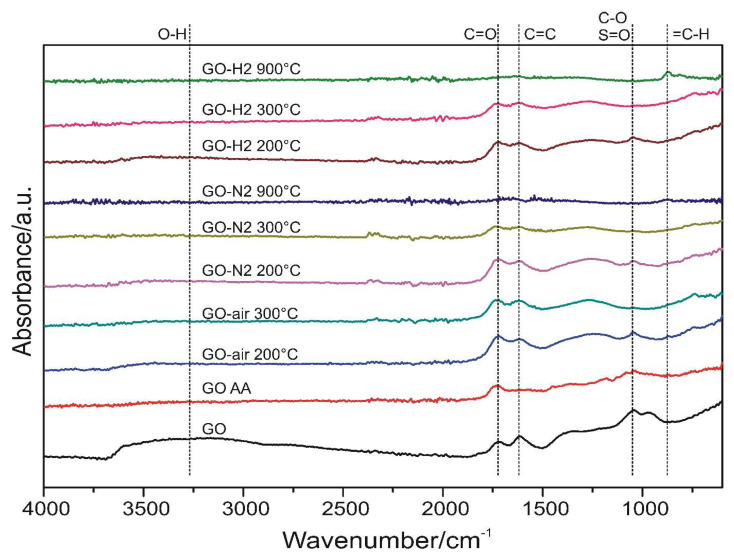
ATR spectra of the samples with the peaks marked.

The ATR FTIR results are shown in Figure 6. The O-H peak of the GO was barely visible after heating to 200 °C, and vanished for GO AA, or when the GO was heated to 300 or 900 °C. The intensity of the C=O peaks at 1750 cm^−1^ decreased slightly when heated to 300 °C, compared to the peaks at 200 °C, and completely disappeared at 900 °C with the further reduction. The C=C stretching at 1650 cm^−1^ showed similar behavior to the previously mentioned C=O stretching, except this peak was not present for the GO AA. The C-O and S=O peak at 1100 cm^−1^ decreased when annealed at 200 °C or was reduced chemically with ascorbic acid, and was not visible after any further heat treatment, in accordance with the XPS measurements. This confirms that at 200 °C, only oxygen-containing functional groups were released from the samples. A significant =C-H peak appeared at 900 °C in hydrogen atmosphere, which suggests, along with the TG/DTA-MS data, the hydrogenation of the aromatic rings.

**Figure 7:**
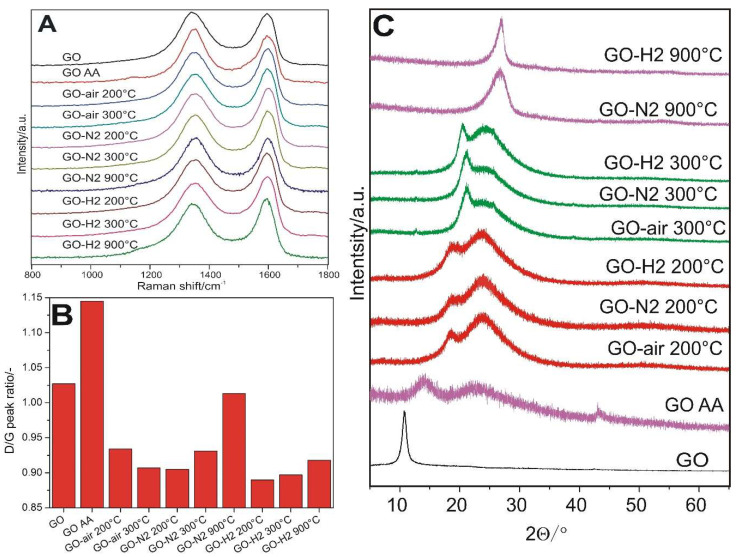
Raman spectra (**A**), the intensity ratios of the carbon D/G peaks (**B**) and the XRD diffractograms (**C**) for the samples.

In the Raman spectra (Figure 7A), there are two characteristic peaks in the carbon, the D (~1350 cm^−1^) and G (~1600 cm^−1^) bands, whose intensity ratio gives information about the regularity of the structure (Figure 7B). Compared to GO, in all cases of thermal reduction, the I_D_/I_G_ ratio was reduced, and the structure became more graphene-like. The I_D_/I_G_ ratio had its minimum when GO was heated at 300 °C in air and at 200 °C in nitrogen and hydrogen. However, then this ratio started to grow when annealing continued, particularly in nitrogen. When the GO was treated chemically with ascorbic acid, the I_D_/I_G_ ratio significantly increased, because the GO plates fragmented, as seen on the SEM and TEM results as well (Figure 3H and Figure 4B).

In the XRD diffractograms in Figure 7C, the characteristic 001 peak of the graphene oxide at 11° shifted to the right with reduction, and two broad, overlapping peaks were visible at 18° and 24° at 200 °C, and at 21° and 25° when heated to 300 °C, which confirmed the formation of reduced graphene oxide sheets. At 900 °C, only one, the 002 peak, remained. The interlayer distance (in Table 2, calculated from the Bragg equation) of the GO (d_001_) decreased when heated to 300 from 200 °C. However, the 002 spacing (d_002_) for reduced graphene oxide, along with the crystallite thickness (D_002_, from the Scherrer equation) and number of graphene layers (N = D_002_/d_002_), was similar at 200 and 300 °C, independently of the atmosphere used for both d_001_ and d_002_. In the case of GO AA, the d_001_ and d_002_ were the highest, and the D_002_ was the lowest, compared to thermally reduced samples, because GO AA was reduced to a lesser extent, and fragmentation occurred during ultrasonication [35,36]. When the GO was heated to 900 °C, the number of graphene layers increased, which is in conjunction with the Raman results.

### 3.2. Application Studies

**Figure 8:**
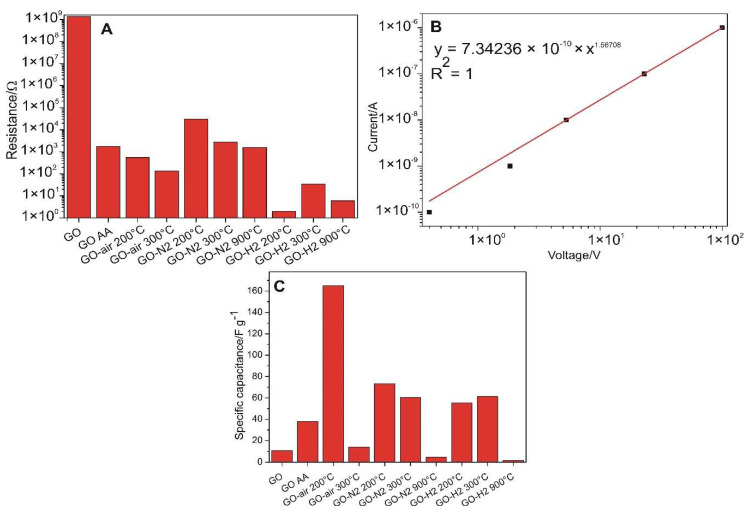
Measured resistance values for the samples (**A**), the current-voltage relationship for the bare GO (**B**) and the specific capacitances calculated from the cyclic voltammograms obtained in 0.5 M H_2_SO_4_ (**C**).

The measured electrical resistance (R) values are shown in Figure 8A. GO had its average resistance value around 1.3 GΩ. It did not behave according to Ohm’s law, as expected, being a semiconductor. The non-linearity can be approximated well by fitting to a power law expression (Figure 8B). The conduction increased by orders of magnitude with the heat treatment. The samples reduced in air at 200 and 300 °C had their resistances below 1 kΩ. In nitrogen, the resistance was higher compared to air, i.e., the R was 30 kΩ when annealed at 200 °C, and became 2.8 and 1.5 kΩ with further heating to 300 and 900 °C, respectively. The higher resistances in nitrogen may result from the fact that, on the one hand, in air atmosphere in the presence of oxygen, the surface groups could decompose more easily, and thus the reduction occurred to a higher extent. On the other hand, nitrogen was incorporated in the structure, as seen from XPS results, and its presence could increase the resistance. For the GO reduced with diluted hydrogen, miniscule resistance (only a few Ωs) was measured independently of the used temperature. This indicates that the reduction process was immensely helped by the hydrogen atmosphere, as the restored graphene structure was a very good conductor. The chemically reduced graphene oxide (GO AA) showed similar resistance to the GO-N2 900 °C sample. These results indicate that the conductivity of GO can be varied by six orders of magnitude, offering a tool to match the electrical properties to the certain applications, e.g., for materials and tools, which need high conductivity, graphene oxide should be reduced in hydrogen atmosphere.

**Figure 9:**
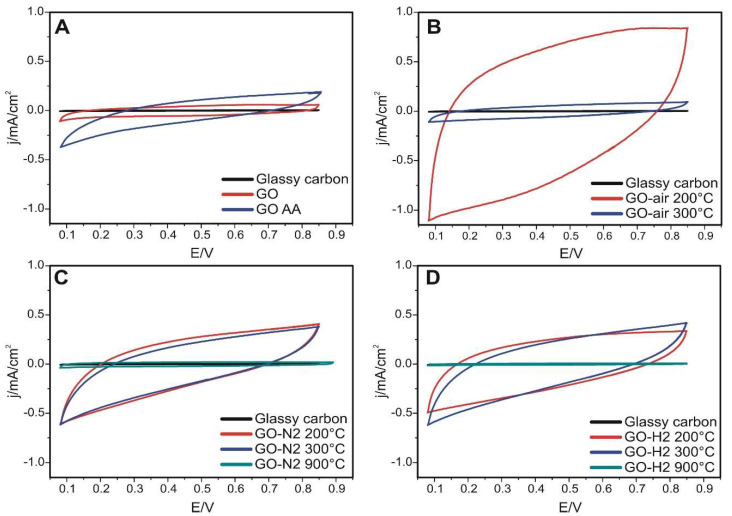
Cyclic voltammetry measurements for the samples in 0.5 M H_2_SO_4_. (**A**) samples GO and GO AA, (**B**) samples in air, (**C**) samples in nitrogen, (**D**) sample in argon/hydrogen.

Cyclic voltammetric curves are shown in Figure 9. The estimated specific capacitances with H_2_SO_4_ (Figure 8C) were calculated from the charge measured between 200 and 700 mV during the positive polarization. The GO sample gave a larger signal than the reference glassy carbon, which increased slightly after reduction with ascorbic acid. In the case of air treatment at 200 °C, the value of the specific capacity increased to the largest of all, as it became 164.9 F g^−1^. Then, in air, it decreased at 300 °C to a value similar as the bare GO, probably due to compaction during the heat treatment. Samples treated at 200 and 300 °C in nitrogen and hydrogen showed similar values; in nitrogen it was slightly higher at 200 °C, while in hydrogen it was larger at 300 °C. The curves of the samples treated at 900 °C hardly differ from the glassy carbon electrode. This suggests that the available electrochemical surface is very small, which may be due to compaction due to the high-temperature heat treatment. In order to obtain a high capacitance of GO materials to be used in supercapacitors, treatment in air atmosphere and at mild temperatures should be used. Our record capacitance (GO-air 200 °C) result corroborates the work of Zhao et al., where a differently prepared graphene oxide treated at 200 °C showed the best performance [16].

**Figure 10:**
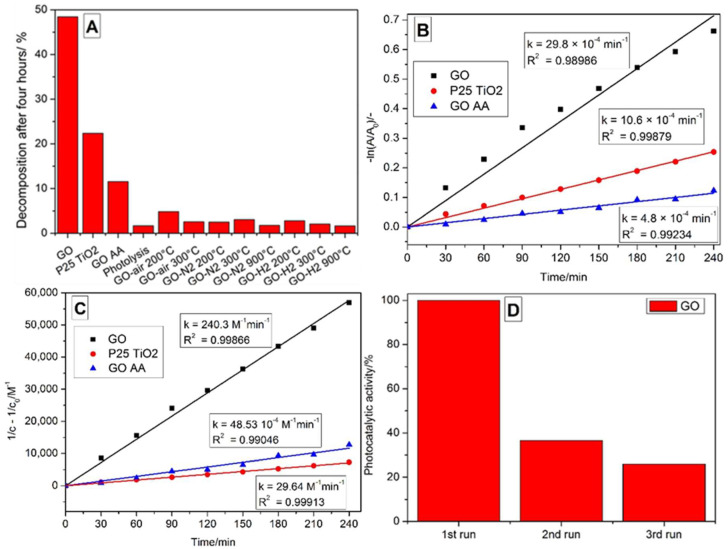
Decomposed amount of methyl orange dye during the photocatalytic experiments (**A**), pseudo first order (**B**) and second order (**C**) linear fitting for the GO, GO AA and P25 TiO_2_ samples, and photocatalytic activity of the GO after repeated measurements (**D**).

The photocatalytic activity of the bare GO is considerable. It decomposed almost half of the methyl orange dye after four hours of illumination (Figure 10A), which was more than two times better performance than the reference P25 titania. This high activity can be explained by the higher specific surface area of the GO compared to the P25, and the enhanced light absorption because of its black color (Figure S7). The reduction decreased the activity in the case of all samples, however, the thermal reduction affected it far more significantly. The reduced activity can be explained by the increased conductivity through the restored graphene-like structure, and the loss of functional groups de-emphasizes the semiconductor characteristics. This process is beneficial when RGO is used in conjunction with a semiconductor oxide because the reduced graphene oxide can act as an electron sink, but the reduction inhibits its innate photocatalytic properties. The difference between the activity of the chemically and thermally reduced GO is related to the regularity of the structure and the number of defects. While during the thermal reduction the samples aggregated because of the restacking, in the case of chemical reduction, the fragmentation caused a higher surface area, less regular structure and was the least reduced sample. Comparing the thermally reduced samples, it can be seen that the lowest activity comes from the 300 °C reduction in hydrogen, while the highest comes from 200 °C reduction in air, but all of them portray considerably lower photocatalytic activity than bare GO. The GO, GO AA and P25 TiO_2_ samples followed a Langmuir–Hinshelwood mechanism during the photocatalysis (Figure 10B), which is indicated by the goodness of fitted line to the assumed pseudo first-order kinetics (Equation (1)). The behavior could be approximated by second-order kinetics (Figure 10C) as well (Equation (2)), with slightly better fitting [37,38,39]. The current and starting concentration of the dye is c and c_0_, respectively, A and A_0_ is the measured and starting absorbance, k is the reaction rate constant and t is time, and the Lambert–Beer law holds true for these concentrations. The repeatability measurements for the GO on Figure 10D show that after consecutive experiments on the same sample, the activity decreases.

−ln(c/c_0_) = −ln(A/A_0_) = k∗t (1)

1/c − 1/c_0_ = k∗t (2)

## 4. Conclusions

By annealing the graphene oxide made with Hummers’ method, at 200 °C, only the oxygen-containing functional groups decompose, while the sulfur-containing ones remain, which are only released when heated above this temperature. From 700 to 900 °C in a partial hydrogen atmosphere, the sample weight increased by 2%, signaling hydrogenation of the aromatic rings, which was confirmed by a relatively high ATR peak for =C-H. The ultrasonication used for the chemical reduction in GO with ascorbic acid fragmented the graphene oxide plates, while the heat treatments resulted in tight structures, which were reflected in the SEM and TEM images. According to EDX, XPS, ATR-FTIR, Raman and XRD measurements, due to the thermal and chemical reduction, the GO transformed into RGO; however, the used reduction parameters strongly affected the composition, functional groups and bonds in the samples.

The electrical resistance measurements show that the conductivity of GO can be varied with the treatment, offering a tool to match its electrical properties to the certain applications. The electrical conductivity increased by orders of magnitude after any reduction, most notably under hydrogen atmosphere. 

The specific capacitances of the GO and RGO samples were estimated from cyclic voltammetric curves. Low temperature reduction (200 °C) in air offers the RGO material with the highest capacitance, with a tempting value (164.9 F g^−1^) to be used in a supercapacitor application. Based on our studies, in order to obtain a high capacitance of GO materials to be used in supercapacitors, treatment of GO in air atmosphere and at mild temperatures should be applied.

According to photocatalysis tests, the bare graphene oxide shows a significant photocatalytic effect in decomposing methyl orange dye under UV light irradiation, more than two times better than the commonly used P25 TiO_2_ reference. Reduction by ascorbic acid impaired the photocatalytic properties and, after thermal treatment, considerably less activity remained because most of the functional groups broke off. According to these, RGO can be used as a beneficial electron sink in composite photocatalysts, but it does not contribute to the photocatalytic activity by itself. 

## Figures and Tables

**Table 1:** Deconvolution of the C1s peak and elemental composition from XPS spectra.

Sample	Deconvolution of C1s Peak/at. %					Elemental Composition/at. %			
	sp2 Graphitic Carbon284.4 eV	sp3 Carbon 285.4 eV	C-O 286.8 eV	C=O 288.4 eV	O-C=O /Loss Feature 290.4 eV	C1s	S2p	O1s	N1s
GO	5.38	44.3	31.8	13.2	5.35	67.2	0.3	32.5	
GO AA	7.73	73.2	7.45	3.63	8.02	67.6		32.4	
GO-air 300 °C	46.3	32.9	3.41	8.28	9.09	80.2	0.15	19.6	
GO-N2 300 °C	48.8	26.9	7.86	5.21	11.2	78.6	0.34	20.3	0.74
GO-H2 300 °C	39.5	39.1	6.85	5.73	8.80	82.6	0.67	16.8	

**Table 2:** Different properties of the samples calculated from XRD measurements.

Sample	d_001_/nm	d_002_/nm	D_002_/nm	N/-
GO	0.81			
GO AA	0.63	0.39	0.62	1.6
GO-air 200 °C	0.47	0.37	2.25	6.0
GO-N2 200 °C	0.47	0.37	2.13	5.7
GO-H2 200 °C	0.48	0.37	2.33	6.2
GO-air 300 °C	0.42	0.37	2.06	5.5
GO-N2 300 °C	0.42	0.37	2.25	6.0
GO-H2 300 °C	0.43	0.36	2.16	6.0
GO-N2 900 °C		0.34	2.58	7.7
GO-H2 900 °C		0.33	5.61	17.0

## Supplementary Materials

The following are available online at https://www.mdpi.com/2079-4991/10/11/2313/s1, Figure S1: Schematic picture of the electrical resistance measurement setup, Figure S2: Spectrum of the UV lamp used for the photocatalytic experiments, Figure S3: XPS survey spectra for five samples, Figure S4: Deconvolution of the C1s peaks for five samples, Figure S5: Deconvolution of the O1s peaks for five samples, Figure S6: N1s peak for sample GO-N2 300°C, Figure S7: UV-Vis reflectance spectra for GO, GO AA and P25 TiO_2_.
