# Supplementary material for: Electric and Photocatalytic Properties of Graphene Oxide Depending on the Degree of Its Reduction
Source: Nanomaterials (Basel). 2020 Nov 22;10(11):2313. doi: 10.3390/nano10112313 (PMC7700189; doi:10.3390/nano10112313)
Supplement: Supplementary file 1 [file nanomaterials-10-02313-s001.pdf]

Supplementary information for the manuscript

Electric and photocatalytic properties of graphene oxide depending on the degree of its reduction

László Péter Bakos[1], Lőrinc Sárvári[1], Krisztina László[2], János Mizsei[3], Zoltán Kónya[4], Gyula Halasi[4], Klára Hernádi[4], Anna Szabó[4], Dániel Berkesi[4], István Bakos[5] and Imre Miklós Szilágyi[1*]

[1]Department of Inorganic and Analytical Chemistry, Budapest University of Technology and Economics, Szent Gellért tér 4., H-1111 Budapest, Hungary
[2]Department of Physical Chemistry and Materials Science, Budapest University of Technology and Economics, Budafoki út 8. F. I. building, H–1111 Budapest, Hungary
[3]Department of Electron Devices, Budapest University of Technology and Economics, H-1117 Budapest, Hungary
[4]Department of Applied and Environmental Chemistry, University of Szeged, Rerrich Béla tér 1., H-6720 Szeged, Hungary
[5]Institute of Materials and Environmental Chemistry, Research Centre for Natural Sciences, Hungarian Academy of Sciences Centre of Excellence, Magyar tudósok körútja 2, H-1117 Budapest, Hungary

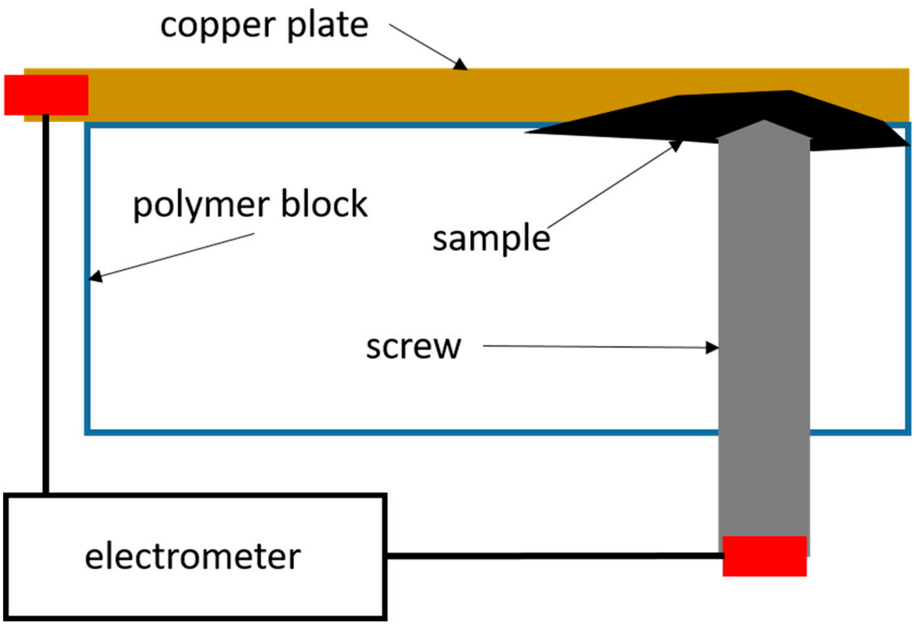

Figure S1 Schematic picture of the electrical resistance measurement setup

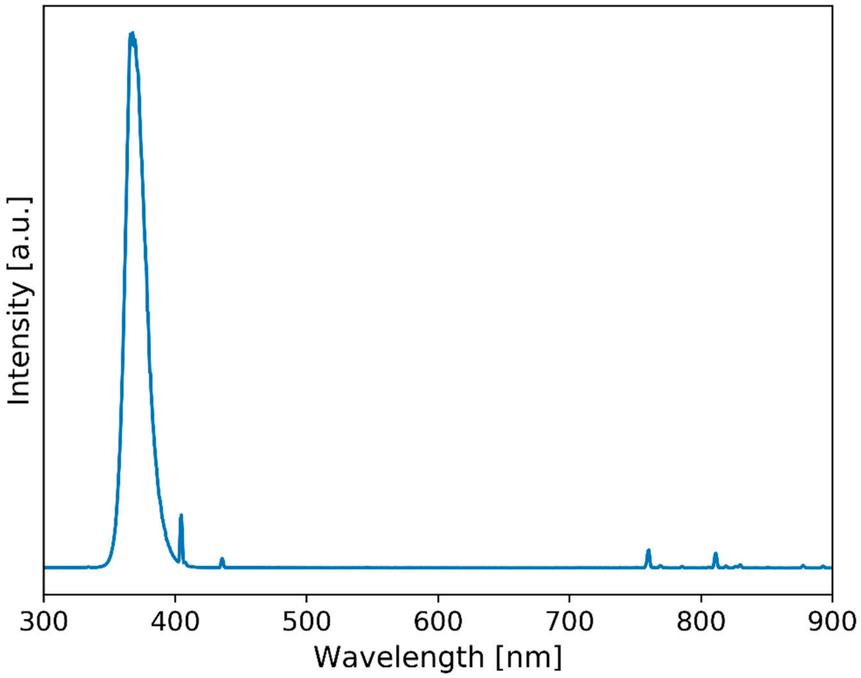

Figure S2 Spectrum of the UV lamp used for the photocatalytic experiments

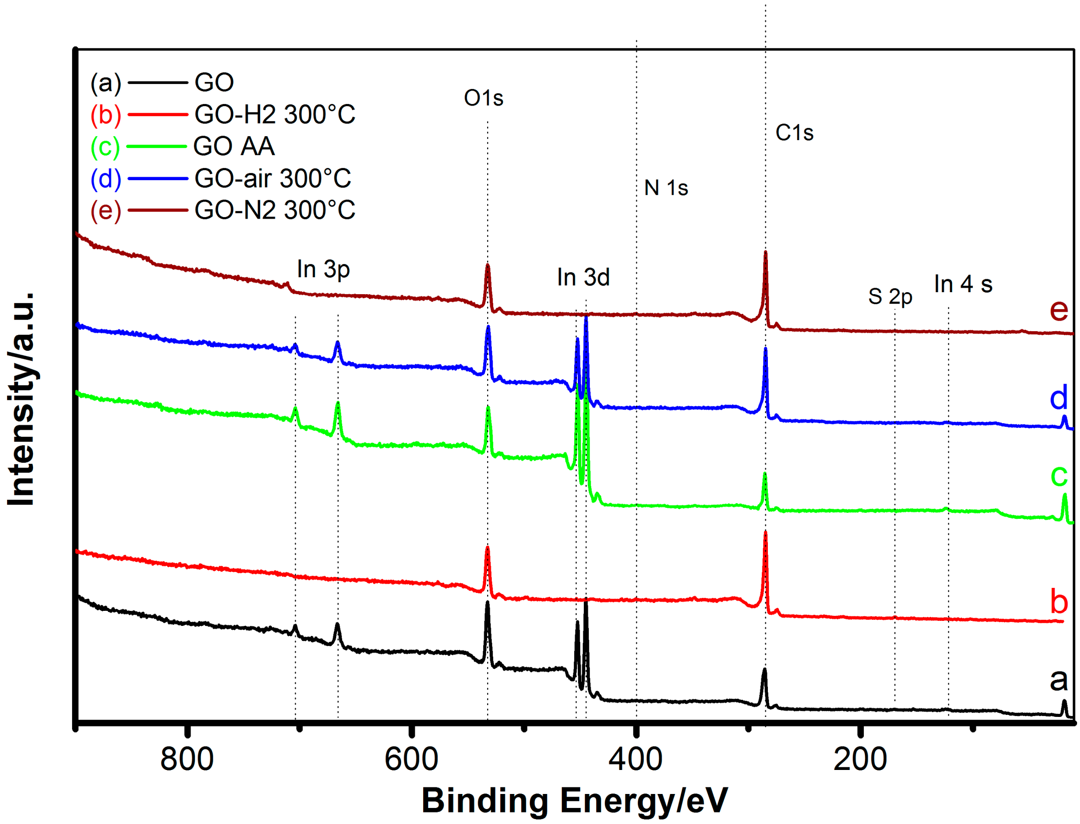

Figure S3 XPS survey spectra for five samples

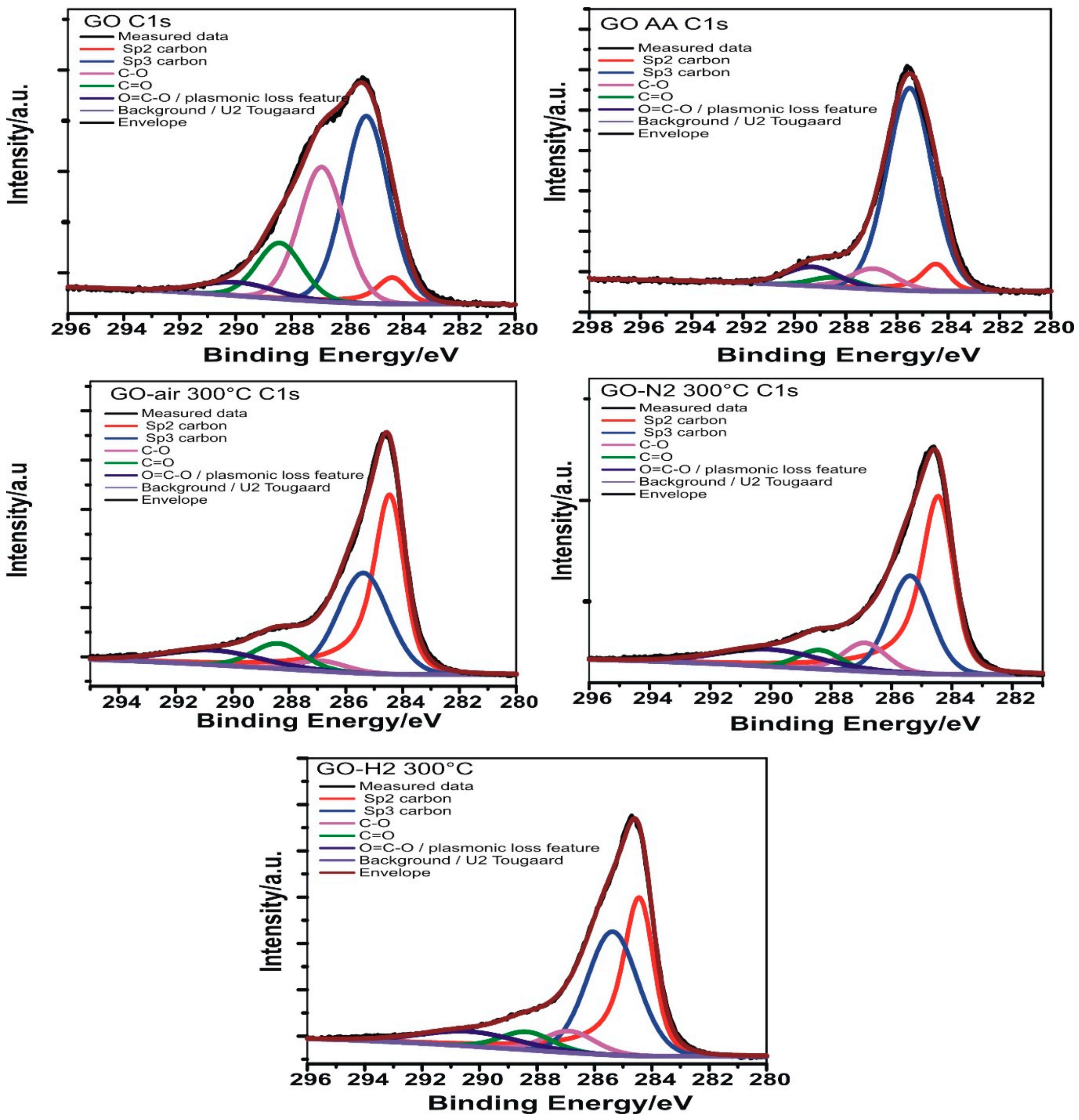

Figure S4 Deconvolution of the C1s peaks for five samples

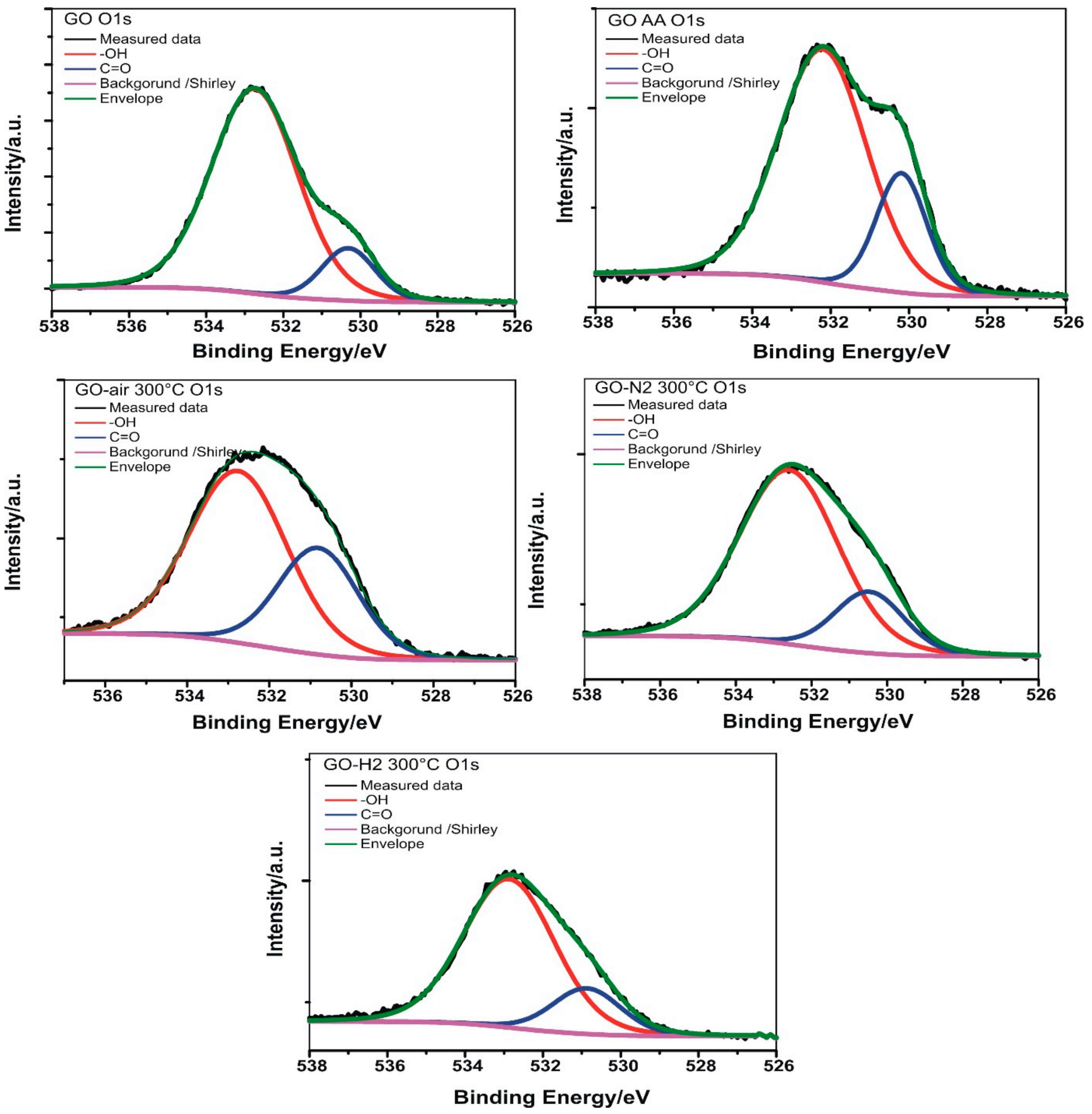

Figure S5 Deconvolution of the O1s peaks for five samples

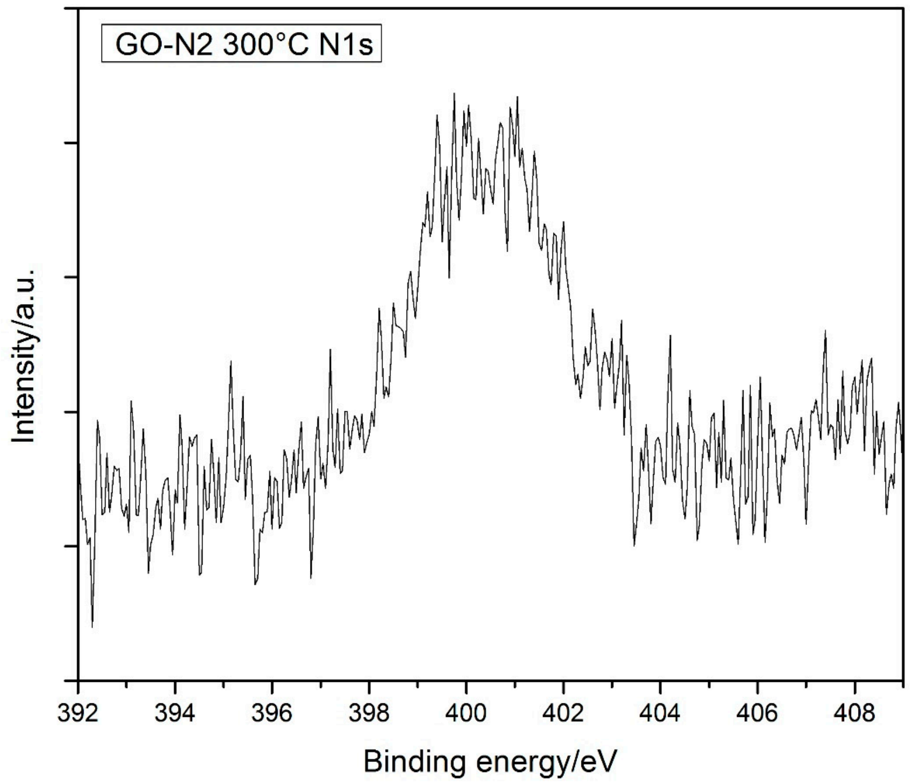

Figure S6 N1s peak for sample GO-N2 300°C

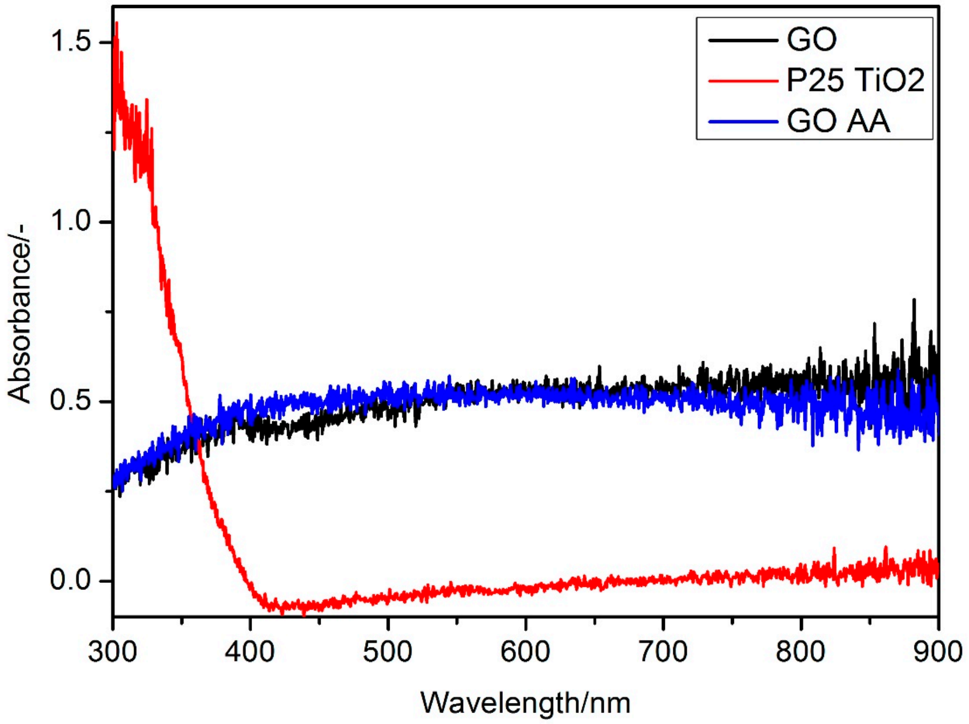

Figure S7 UV-Vis reflectance spectra for GO, GO AA and P25 TiO_2
